# Pulmonary Alveolar Microlithiasis: A Rare Case Report 

**Published:** 2013-09

**Authors:** Kaleem Ahmad, Mukesh Kumar Gupta, Kanchan Dhungel, Panna Lal Sah, Sajid Ansari, Raj Kumar Rauniyar

**Affiliations:** Department of Radiodiagnosis, B.P. Koirala Institute of Health Sciences, Dharan, Nepal

**Keywords:** Pulmonary alveolar microlithiasis, Calcification, Computed tomography

## Abstract

Pulmonary alveolar microlithiasis is an uncommon infiltrative pulmonary disease characterized by deposition of microliths in the alveoli. We present the case of a young adult with complaints of shortness of breath on exertion. Chest radiograph showed innumerable small, dense nodules, diffusely involving both the lungs - predominantly in the lower zones. High-resolution CT scan illustrated widespread intra-alveolar microliths, diffuse ground-glass attenuation areas, septal thickening, and black pleural lines - predominantly in the basal regions. Transbronchial biopsy confirmed the diagnosis.

## Introduction

Pulmonary alveolar microlithiasis (PAM) is a rare pulmonary disease in which there is deposition of calcium phosphate microliths within the alveoli.^1^ This rare entity was first brought into notice by Friedrich in 1856 and subsequently by Harbitz in 1918. The etiology of PAM is unclear; however, one of the probable causes may be isolated inborn errors of calcium metabolism and it could be regarded as hereditary autosomal recessive lung disease.^2^ Mutation in type IIb sodium-phosphate cotransporter gene (SCL34A2 gene) has recently been described by some authors in the pathogenesis of PAM.^3^ The usual age of the presentation of PAM is between 20 and 40 years, and the majority of the cases are diagnosed incidentally on plain radiograph. PAM has variable clinical manifestations, and patients may remain asymptomatic for a longer time, although crackles, clubbing, cyanosis, and signs of respiratory failure may be observed during the late course of the disease. Patients eventually develop dyspnea on exertion, which limits their physical activity, and in the advance stage of the disease, respiratory failure and cor pulmonale ensues. The pulmonary function test demonstrates restrictive lung disease, which results in cardio-respiratory failure. 

Herein, we report the case of a 27-year-old man with suspicion of PAM on the basis of chest radiograph, which was confirmed by high-resolution computed tomographic (HRCT) scan and transbronchial biopsy.

## Case Presentation

A 27-year-old man presented with complaints of shortness of breath on exertion and dry cough of 2 years’ duration. He had been a carpenter by profession for the last 5 years. There was no history of fever, chest pain, hemoptysis, or weight loss. He was a non-smoker and had no pulmonary disease or significant family history. On auscultation, there were wheezes and coarse crackles bilaterally. Cardiac auscultation was normal, and no cyanosis/clubbing/peripheral edema was observed. The routine blood examination was found to be normal, and the pulmonary function tests showed mild restrictive lung disease.

**Figure 1:**
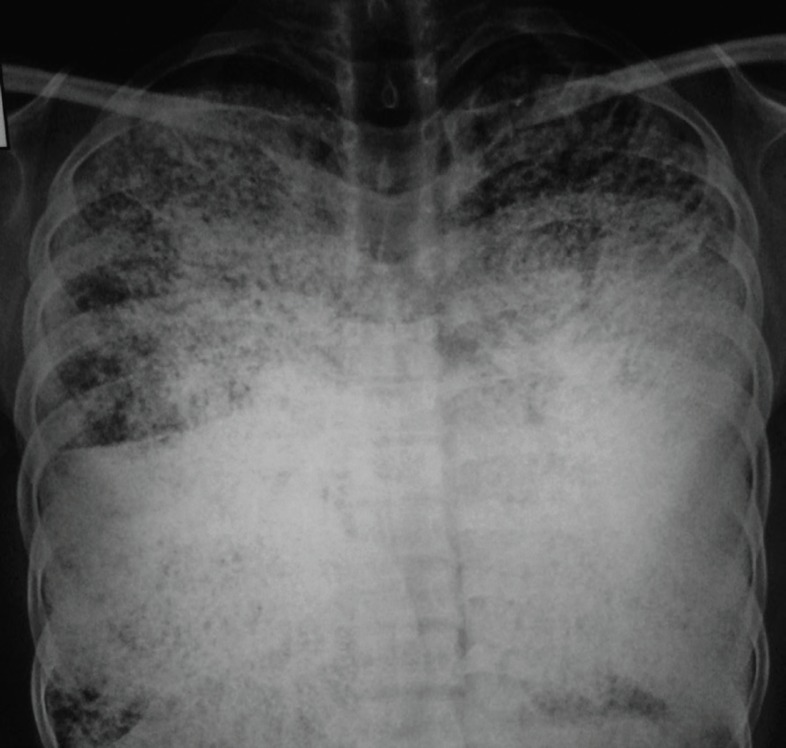
This chest radiograph (posteroanterior view) shows innumerable widespread, small, dense nodules, diffusely involving both lungs-predominantly in the basal regions - with obscuration of the mediastinal, cardiac, and diaphragmatic borders. A few fibrotic strands can be detected in bilateral lungs

Chest radiograph posteroanterior view (figure 1) revealed the presence of innumerable widespread, small, dense nodules-diffusely involving both the lungs-predominantly in the basal regions with obscuration of the mediastinal, cardiac, and diaphragmatic borders. A few fibrotic strands were also seen. 

**Figure 2:**
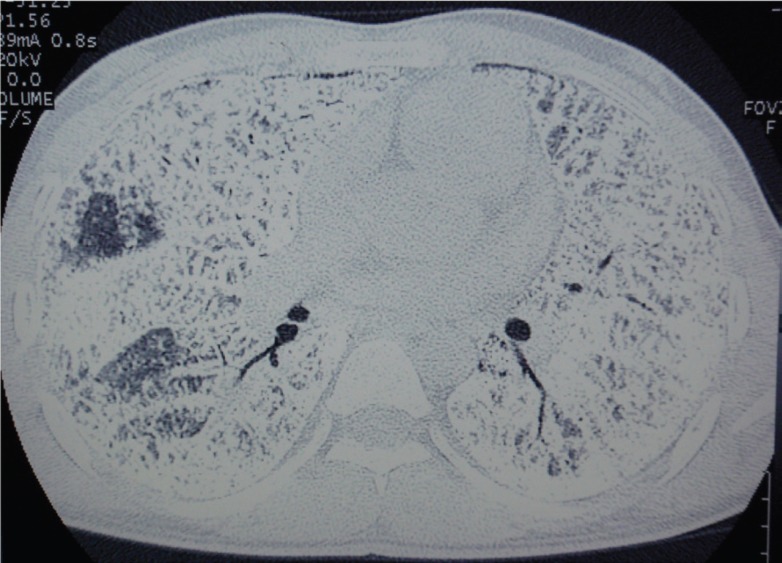
This high-resolution computed tomogram chest demonstrates diffuse intra-alveolar opacities of calcific density in bilateral lung parenchyma, septal thickening, and black pleural lines along with calcification along the interlobar septa and subpleural regions

**Figure 3a and 3b:**
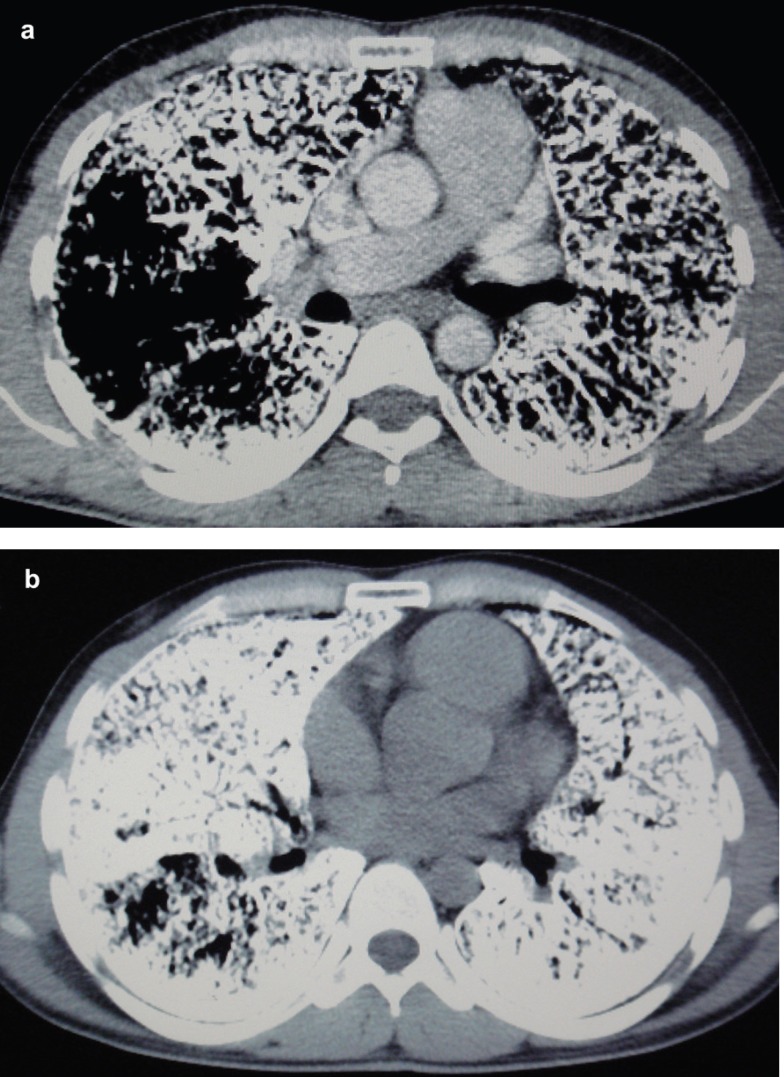
This multidetector computed tomogram chest (mediastinal window) illustrates diffuse ground-glass opacities in bilateral lung parenchyma, septal thickening, and calcification along the interlobar septa and subpleural regions with black pleural lines

HRCT of the chest (figure 2) showed the presence of widespread nodular intra-alveolar opacities of calcific density with diffuse ground-glass attenuation, more pronounced in the lower pulmonary regions. Calcifications were seen along the interlobar septa and subpleural regions. There was also evidence of septal thickening. Subpleural cysts, black pleural lines, and a few fibrotic changes were also noticed. These features were consistent with the diagnosis of PAM. Multidetector computed tomography (MDCT) of chest (mediastinal window, figures 3a and 3b) revealed diffuse ground-glass opacities in bilateral lung parenchyma, septal thickening, and calcification along the interlobar septa and subpleural regions with black pleural lines.

The patient was advised to undergo bronchoalveolar lavage and lung biopsy. Fiberoptic bronchoscopy was done along with bronchoalveolar lavage, in which microliths were observed. The lavage fluid was not suggestive of tuberculosis or fungi. Transbronchial biopsy was performed, which revealed concentric laminated microliths in the alveoli along with thickened interstitial septa, confirming the diagnosis of PAM.

## Discussion

The incidence of PAM is worldwide; however, approximately one-quarter of the patients are from Turkey - having almost equal male and female sex predilection ^4^ Mostly, patients affected with this disease are asymptomatic and are diagnosed incidentally on imaging. Patients become symptomatic usually with the advancement of the disease. Non-productive cough and dyspnea on exertion are the common symptoms; nevertheless, in the later course of the disease - respiratory insufficiency, cor pulmonale, and even death may occur.^5^

On chest radiograph, numerous sand-like microliths or calcispherites are seen diffusely scattered in bilateral lung fields - predominantly in the lower two-thirds of the lungs - obscuring the diaphragmatic, mediastinal, and cardiac borders. The propensity of the disease for the lung bases is probably due to the larger volume of the lower lobes. Bullae in the lung apices, a zone of hyperlucency between the lung parenchyma and the ribs (known as a black pleural line), and calcification in the pleura could be the other manifestations. The pattern of calcification may be uniform or may show coarsely linear nodulations. Also, reticulations and septal lines can occasionally be seen on chest radiograph.^6^

For the evaluation of PAM, HRCT is preferred with thin collimation axial scans and image reconstruction with a high-resolution algorithm. Minimal morphological changes of the lung parenchyma which are not well evaluated on radiography or with other CT techniques can be detected by HRCT. HRCT chest reveals intra-alveolar calcifications (microliths), manifesting as micronodular or ground-glass opacities along with superimposed septal thickening - i.e. crazy-paving pattern - predominantly in the postero-basal regions along the bronchovascular bundles and subpleural regions.^7^ The black pleural lines can be confused due to thin-walled subpleural cysts on HRCT.

There are several diffuse lung diseases with pulmonary calcifications which might be included in the differential diagnosis of PAM such as pulmonary alveolar proteinosis, amyloidosis, metastatic pulmonary calcification, pulmonary vascular diseases, hyperparathyroidism, previous DNA virus infection, and chronic renal failure.^8^

Although PAM can be easily diagnosed by bronchoalveolar lavage,^9^ bronchoalveolar lavage and sputum examination for the presence of microliths are non-specific for the diagnosis of PAM in as much as microliths can also be found in patients with tuberculosis and chronic obstructive pulmonary disease.^10^ Confirmatory diagnosis can be established by open lung or transbronchial biopsy, showing intra-alveolar calcospherites, which in turn represent laminated calcium phosphate concretions.

Currently, there is no specific medical therapy for PAM. And it is also deserving of note that the majority of PAM patients suffer from respiratory insufficiency and the only option left for them is lung transplantation, which can relatively improve respiratory insufficiency.^11^

Novelty of the case described in the present study is that it presents a rare, chronic lung disease, the likes of which have been few and far between in the existing literature. Occupational lung diseases such as allergies, bronchitis, bronchial asthma, and asbestosis have been previously reported among carpenters, but there has been no report on pulmonary alveolar microlithiasis in carpenters. It can, therefore, be concluded that PAM was unrelated to the profession of carpentry in our patient.

## Conclusion

PAM is a rare lung disease and should be considered in the differential diagnosis of diffuse parenchymal disease of chest. HRCT should always be performed as it reveals the characteristic patterns of PAM; however, confirmatory diagnosis is established by transbronchial or open lung biopsy. There is no specific treatment for PAM; nonetheless, lung transplantation can provide improvement in respiratory insufficiency. 
